# Supplementary material for: Association of Serum Vitamin B6 with All-Cause and Cause-Specific Mortality in a Prospective Study
Source: Nutrients. 2021 Aug 27;13(9):2977. doi: 10.3390/nu13092977 (PMC8472743; doi:10.3390/nu13092977)
Supplement: Supplementary file 1 [file nutrients-13-02977-s001.zip › nutrients-1300998-supplementary.pdf]

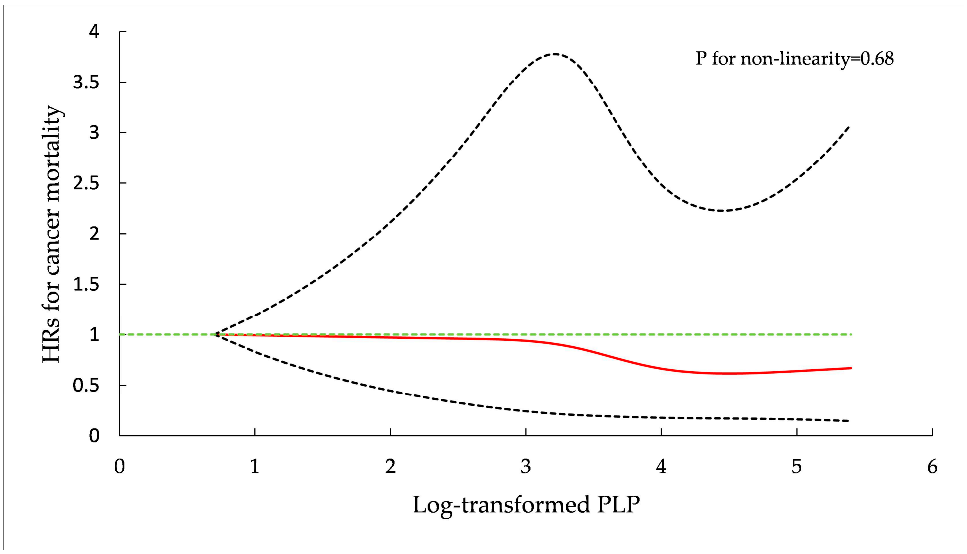

Figure S1. Association of pyridoxal 5′-phosphate (PLP) with cancer mortality in restricted cubic regression with four knots (5th, 35th, 65th, 95th) in NHANES from 2005 to 2010.

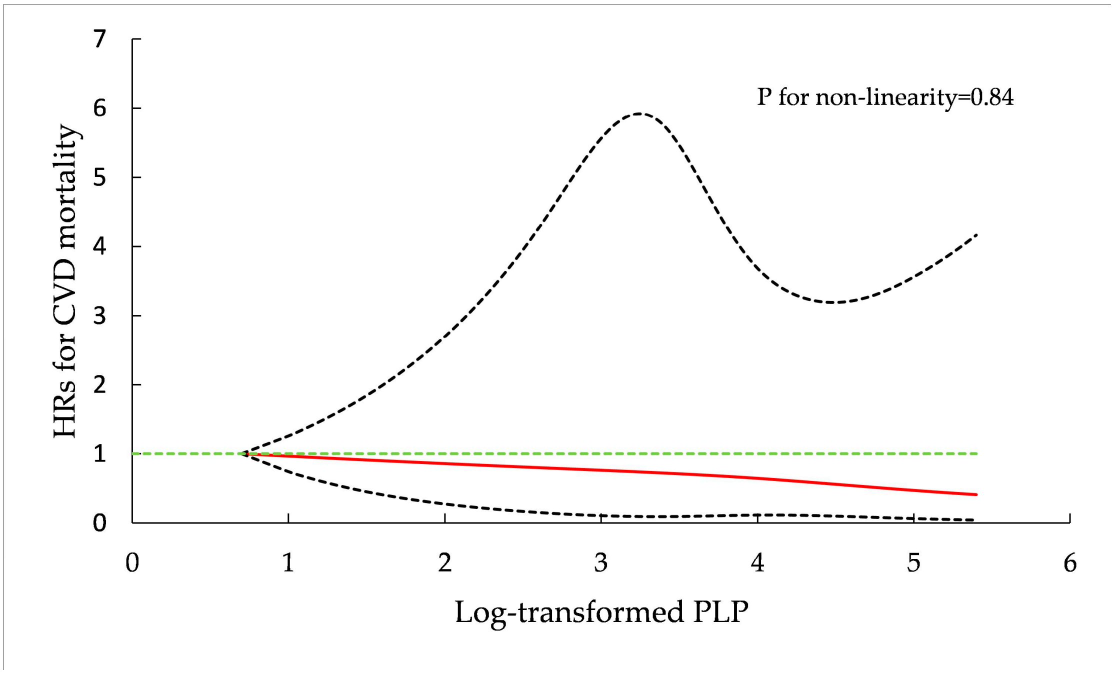

Figure S2: Association of pyridoxal 5′-phosphate (PLP) with cardiovascular disease (CVD) mortality in restricted cubic regression with four knots (5th, 35th, 65th, 95th) in NHANES from 2005 to 2010.

Table S1. Associations between serum vitamin B6 and all-cause mortality among adults from NHANES.

Variable	HR (95%CI)		
	Model 1	Model 2	Model 3
Vitamin B6 status (reference, Sufficient)			
Deficient	1.83(1.55,2.15)	1.50(1.28,1.75)	1.37(1.17,1.60)

Insufficient	1.37(1.17,1.60)	1.20(1.04,1.40)	1.19(1.02,1.38)
Age (reference, <60 years)			
>=60 years	9.60(8.08,11.41)	7.38(6.10,8.93)	6.42(5.31,7.76)
Sex (reference, male)			
Female	0.71(0.62,0.81)	0.56(0.48,0.66)	0.53(0.45,0.62)
Race/ethnicity (reference, Hispanic)			
Non-Hispanic White		1.58(1.26,1.98)	1.51(1.21,1.88)
Non-Hispanic Black		1.30(1.03,1.63)	1.21(0.97,1.52)
Non-Hispanic other		1.16(0.70,1.94)	1.04(0.86,1.73)
Educational level (reference, less than high school)			
High school or equivalent		0.80(0.72,0.88)	0.86(0.77,0.95)
College or above		0.64(0.53,0.76)	0.75(0.62,0.91)
Marital Status (reference, Married or living with partner)			
Widowed, divorced, or separated		2.03(1.70,2.42)	1.99(1.66,2.39)
Never married		0.91(0.67,1.25)	0.96(0.69,1.33)
Family income-poverty ratio (reference, 0.0-1.0)			
1.1-3.0		0.95(0.8,1.14)	1.03(0.86,1.23)
>3.0		0.51(0.4,0.65)	0.62(0.48,0.80)
BMI，kg/m2 (reference, normal weight or underweight [<25.0])			
Pre-obese [25.0-29.9]			0.86(0.69,1.07)
Obese [⩾30]			0.71(0.61,0.82)
Drinking status (reference, never drinker)			
Ever drinker			1.18(0.89,1.58)
Current drinker			0.62(0.49,0.79)
Smoking Status (reference, never smoker)			
Ever smoker			1.20(0.98,1.45)
Current smoker			1.23(1.03,1.48)
Physical activity (reference, <150min MVPA/week)			
⩾150min MVPA/week			0.66(0.57,0.77)

Table S2. Associations between serum vitamin B6 and cancer mortality among adults from NHANES.

Variable	HR (95%CI)		
	Model 1	Model 2	Model 3
Vitamin B6 status (reference, Sufficient)			
Deficient	1.72(1.19,2.49)	1.43(1.00,2.03)	1.22(0.85,1.74)
Insufficient	1.61(1.11,2.32)	1.43(0.98,2.11)	1.38(0.95,2.02)
Age (reference, <60 years)			
>=60 years	6.63(4.56,9.64)	5.01(3.34,7.50)	4.46(3.00,6.63)
Sex (reference, male)			
Female	0.57(0.43,0.75)	0.50(0.37,0.68)	0.51(0.35,0.73)
Race/ethnicity (reference, Hispanic)			
Non-Hispanic White		1.42(0.97,2.08)	1.26(0.85,1.86)
Non-Hispanic Black		1.70(1.16,2.49)	1.47(0.99,2.19)

Non-Hispanic other		0.81(0.28,2.36)	0.69(0.23,2.08)
Educational level (reference, less than high school)			
High school or equivalent		0.68(0.49,0.96)	0.75(0.54,1.04)
College or above		0.58(0.39,0.88)	0.71(0.47,1.07)
Marital Status (reference, Married or living with partner)			
Widowed, divorced, or separated		1.51(1.10,2.08)	1.49(1.08,2.06)
Never married		0.42(0.21,0.87)	0.47(0.23,0.95)
Family income-poverty ratio (reference, 0.0-1.0)			
1.1-3.0		1.12(0.72,1.74)	1.19(0.77,1.86)
>3.0		0.70(0.43,1.15)	0.86(0.52,1.43)
BMI，kg/m2 (reference, normal weight or underweight [<25.0])			
Pre-obese [25.0-29.9]			0.76(0.52,1.10)
Obese [⩾30]			0.84(0.61,1.17)
Drinking status (reference, never drinker)			
Ever drinker			1.64(0.89,3.00)
Current drinker			0.69(0.41,1.15)
Smoking Status (reference, never smoker)			
Ever smoker			1.45(0.95,2.23)
Current smoker			1.61(1.14,2.28)
Physical activity (reference, <150min MVPA/week)			
⩾150min MVPA/week			0.81(0.57,1.15)

Table S3. Associations between serum vitamin B6 and CVD mortality among adults from NHANES.

Variable	HR (95%CI)		
	Model 1	Model 2	Model 3
Vitamin B6 status (reference, Sufficient)			
Deficient	1.65(1.18,2.31)	1.31(0.93,1.85)	1.26(0.89,1.77)
Insufficient	1.29(0.78,2.12)	1.07(0.66,1.73)	1.07(0.65,1.78)
Age (reference, <60 years)			
>=60 years	15.17(9.64,23.88)	11.61(6.89,19.56)	10.05(5.86,17.23)
Sex (reference, male)			
Female	0.46(0.37,0.63)	0.33(0.23,0.46)	0.29(0.22,0.40)
Race/ethnicity (reference, Hispanic)			
Non-Hispanic White		1.97(1.28,3.04)	1.98(1.26,3.12)
Non-Hispanic Black		1.53(0.99,2.38)	1.51(0.95,2.41)
Non-Hispanic other		1.80(0.60,5.37)	1.77(0.57,5.55)
Educational level (reference, less than high school)			
High school or equivalent		0.90(0.63,1.30)	0.96(0.67,1.37)
College or above		0.69(0.46,1.05)	0.79(0.51,1.20)
Marital Status (reference, Married or living with partner)			

Widowed, divorced, or separated	2.66(1.88,3.78)	2.61(1.80,3.80)
Never married	1.20(0.70,2.05)	1.23(0.71,2.13)
Family income-poverty ratio (reference, 0.0-1.0)		
1.1-3.0	1.07(0.72,1.60)	1.12(0.76,1.66)
>3.0	0.41(0.26,0.66)	0.47(0.30,0.74)
BMI，kg/m2 (reference, normal weight or underweight [<25.0])		
Pre-obese [25.0-29.9]		0.97(0.55,1.69)
Obese [≥30]		0.79(0.56,1.10)
Drinking status (reference, never drinker)		
Ever drinker		0.95(0.64,1.43)
Current drinker		0.69(0.41,1.15)
Smoking Status (reference, never smoker)		
Ever smoker		1.05(0.74,1.50)
Current smoker		0.96(0.58,1.58)
Physical activity (reference, <150min MVPA/week)		
≥150min MVPA/week		0.59(0.39,0.90)

Table S4: HRs (95% CIs) of all-cause and specific cause mortality according to serum vitamin B6 concentrations after excluding participants dying with 2 years of follow up in NHANES from 2005 to 2010.

	Vitamin B6 status		
	Deficient	Insufficient	Sufficient
All-cause mortality			
Number of deaths/total	214/1804	197/2110	592/8035
Model 1	1.60(1.32,1.93)	1.36(1.12,1.65)	1.00
Model 2	1.32(1.10,1.59)	1.21(1.00,1.45)	1.00
Model 3	1.19(0.99,1.42)	1.18(0.98,1.42)	1.00
Cancer mortality			
Number of deaths	48	49	136
Model 1	1.44(0.97,2.14)	1.49(0.99,2.25)	1.00
Model 2	1.21(0.82,1.78)	1.34(0.88,2.06)	1.00
Model 3	0.98(0.66,1.46)	1.27(0.82,1.95)	1.00
CVD mortality			
Number of deaths	29	38	120
Model 1	1.28(0.85,1.93)	1.33(0.75,2.38)	1.00
Model 2	1.01(0.66,1.56)	1.11(0.63,1.94)	1.00
Model 3	0.94(0.63,1.42)	1.10(0.60,2.00)	1.00

Model 1: adjusted for age (<60 years; ≥60 years) and sex (male; female). Model 2: further adjusted (from Model 1) for race/ ethnicity (Hispanic; non-Hispanic white; non-Hispanic black; other non-Hispanic), marital status (married or living with partner; widowed, divorced, or separated; never married), education level (less than high school; high school or equivalent; college or above), family income-poverty ratio (0–1.0; 1.1–3.0; >3.0). Model 3: further adjusted (from Model 2) for BMI (normal weight or underweight [<25kg/m^2], pre-obese [25-29.9kg /m^2], obese [≥30kg/m^2]), smoking status (never smoker; former smoker; current smoker), drinking status (never drinker; former drinker; current drinker), physical activity (<150 min MVPA/week; ≥150 min MVPA/week).

Table S5: HRs (95% CIs) of all-cause and specific cause mortality according to serum vitamin B6 concentrations after further adjustment for estimated glomerular filtration rate (eGFR) in NHANES from 2005 to 2010.

	vitamin B6 status		
	Deficient	Insufficient	Sufficient
All-cause mortality			
Number of deaths/total	296/1886	244/2157	704/8147
Model 1	1.84(1.56,2.17)	1.36(1.17,1.58)	1.00
Model 2	1.52(1.30,1.77)	1.20(1.03,1.39)	1.00
Model 3	1.38(1.18,1.62)	1.18(1.02,1.38)	1.00
Cancer mortality			
Number of deaths	67	66	161
Model 1	1.71(1.18,2.49)	1.60(1.10,2.32)	1.00
Model 2	1.42(0.99,2.03)	1.43(0.98,2.11)	1.00
Model 3	1.21(0.83,1.75)	1.38(0.94,2.02)	1.00
CVD mortality			
Number of deaths	47	45	143
Model 1	1.66(1.17,2.34)	1.20(0.75,1.93)	1.00
Model 2	1.32(0.93,1.87)	1.00(0.63,1.59)	1.00
Model 3	1.26(0.88,1.79)	1.00(0.62,1.62)	1.00

Model 1: adjusted for age (<60 years; ≥60 years) and sex (male; female). Model 2: further adjusted (from Model 1) for race/ ethnicity (Hispanic; non-Hispanic white; non-Hispanic black; other non-Hispanic), marital status (married or living with partner; widowed, divorced, or separated; never married), education level (less than high school; high school or equivalent; college or above), family income-poverty ratio (0–1.0; 1.1–3.0; >3.0). Model 3: further adjusted (from Model 2) for BMI (normal weight or underweight [<25kg/m^2], pre-obese [25-29.9kg /m^2], obese [≥30kg/m^2]), smoking status (never smoker; former smoker; current smoker), drinking

status (never drinker; former drinker; current drinker), physical activity (<150 min MVPA/week; ≥150 min MVPA/week), eGFR (≥60ml/min·1.73 m^2; <60ml/min·1.73 m^2).

Table S6: HRs (95% CIs) of all-cause and specific cause mortality according to tertiles of serum pyridoxal 5′-phosphate (PLP) concentrations in NHANES from 2005 to 2010.

	PLP status (nmol/L)		
	≤31.1	31.2-57.3	≥57.4
All-cause mortality			
Number of deaths/total	543/4091	326/4043	375/4056
Model 1	1.66(1.42,1.92)	1.13(0.95,1.34)	1
Model 2	1.35(1.17,1.56)	1.03(0.85,1.24)	1
Model 3	1.28(1.10,1.48)	1.02(0.85,1.22)	1
Cancer mortality			
Number of deaths	133	90	71
Model 1	2.05(1.40,3.00)	1.58(1.05,2.39)	1
Model 2	1.71(1.15,2.55)	1.45(0.94,2.23)	1
Model 3	1.55(1.05,2.31)	1.43(0.93,2.19)	1
CVD mortality			
Number of deaths	95	70	70
Model 1	1.85(1.27,2.71)	1.59(1.09,2.3)	1
Model 2	1.46(1.00,2.14)	1.44(0.99,2.1)	1
Model 3	1.44(0.97,2.16)	1.45(1.00,2.1)	1

Model 1: adjusted for age (<60 years; ≥60 years) and sex (male; female). Model 2: further adjusted (from Model 1) for race/ ethnicity (Hispanic; non-Hispanic white; non-Hispanic black; other non-Hispanic), marital status (married or living with partner; widowed, divorced, or separated; never married), education level (less than high school; high school or equivalent; college or above), family income-poverty ratio (0–1.0; 1.1–3.0; >3.0). Model 3: further adjusted (from Model 2) for BMI (normal weight or underweight [<25kg/m2], pre-obese [25-29.9kg /m2], obese [≥30kg/m2]), smoking status (never smoker; former smoker; current smoker), drinking status (never drinker; former drinker; current drinker), physical activity (<150 min MVPA/week; ≥150 min MVPA/week)
